# Prolonged ursodeoxycholic acid administration reduces acute ischaemia-induced arrhythmias in adult rat hearts

**DOI:** 10.1038/s41598-020-72016-4

**Published:** 2020-09-17

**Authors:** Elisa Ferraro, Lidia Pozhidaeva, David S. Pitcher, Catherine Mansfield, Jia Han Benjamin Koh, Catherine Williamson, Oleg Aslanidi, Julia Gorelik, Fu Siong Ng

**Affiliations:** 1 National Heart and Lung Institute, Imperial College London, London, UK; 2 School of Biomedical Engineering and Imaging Science, King’s College London, London, UK; 3 Department of Women and Children’s Health, King’s College London, London, UK

**Keywords:** Myocardial infarction, Arrhythmias

## Abstract

Acute myocardial ischaemia and reperfusion (I–R) are major causes of ventricular arrhythmias in patients with a history of coronary artery disease. Ursodeoxycholic acid (UDCA) has previously been shown to be antiarrhythmic in fetal hearts. This study was performed to investigate if UDCA protects against ischaemia-induced and reperfusion-induced arrhythmias in the adult myocardium, and compares the effect of acute (perfusion only) versus prolonged (2 weeks pre-treatment plus perfusion) UDCA administration. Langendorff-perfused adult Sprague–Dawley rat hearts were subjected to acute regional ischaemia by ligation of the left anterior descending artery (10 min), followed by reperfusion (2 min), and arrhythmia incidence quantified. Prolonged UDCA administration reduced the incidence of acute ischaemia-induced arrhythmias (*p* = 0.028), with a reduction in number of ventricular ectopic beats during the ischaemic phase compared with acute treatment (10 ± 3 vs 58 ± 15, *p* = 0.036). No antiarrhythmic effect was observed in the acute UDCA administration group. Neither acute nor prolonged UDCA treatment altered the incidence of reperfusion arrhythmias. The antiarrhythmic effect of UDCA may be partially mediated by an increase in cardiac wavelength, due to the attenuation of conduction velocity slowing (*p* = 0.03), and the preservation of Connexin43 phosphorylation during acute ischaemia (*p* = 0.0027). The potential antiarrhythmic effects of prolonged UDCA administration merit further investigation.

## Introduction

Ventricular arrhythmias occur commonly in patients with coronary artery disease, with acute myocardial infarction (MI) being a major cause. Malignant ventricular arrhythmias arising in the setting of acute ischaemia are due to a number of related factors, including diastolic Ca^2+^ overload, intracellular acidosis, and accumulation of amphipathic lipid metabolites, all of which interact to slow conduction velocity (CV), alter excitability and refractoriness in a spatially heterogeneous manner, and generate spontaneous electrical activity^1–3^. Conduction slowing in acutely ischaemic myocardium is in part due to acute gap junction uncoupling, and Connexin43 (Cx43) dephosphorylation contributes to this process^4,5^. Additionally, reperfusion ventricular arrhythmias can occur after restoration of blood flow to previously-ischaemic myocardium, with the mechanisms thought to be due in part to the heterogenous restoration of action potentials (APs) in the first few seconds of reperfusion^6–8^. Despite decreasing mortality from acute MI in the past few decades, novel therapeutic strategies are still needed to reduce the incidence of potentially lethal ventricular arrhythmias induced by acute ischaemia–reperfusion (I–R)^9^.

Within the human bile acid pool, ursodeoxycholic acid (UDCA) is a secondary bile acid currently used clinically to treat hepato-biliary diseases^10^. In vitro studies and clinical trials have proven its beneficial effects in neurodegenerative diseases, alongside a protective role from retinal degeneration, glaucoma and cataracts^11,12^. Relevant to the present study, previous findings have highlighted UDCA’s beneficial properties in the heart: a number of pre-clinical and clinical studies have shown the capacity of UDCA pre-treatment to reduce the infarct size in I–R, to improve ventricular diastolic pressure and re-equilibrate hemodynamic parameters in the ischaemic adult heart, and to improve peripheral blood flow and markers of liver function in patients with chronic heart failure^13–15^. Using an in vitro rat model of the cholestatic fetal heart, we previously showed that UDCA protects against ventricular conduction slowing and arrhythmias by depolarizing myofibroblasts on neonatal rat cardiomyocytes co-cultures^16–18^. The antiarrhythmic effect of UDCA were also attributed to its capacity to target T-type calcium currents (*I*_Ca,T_), exclusively expressed in fetal hearts^19^. Although antiarrhythmic effects have been demonstrated to the fetal heart, though no evidence yet exists for adult hearts.

In this study, we hypothesised that UDCA also exerts antiarrhythmic effects in adult myocardium. We compared the effects of acute versus prolonged UDCA administration on acute regional ischaemia-induced and reperfusion-induced arrhythmia in isolated Langendorff-perfused rat hearts. We systematically investigated the capacity of UDCA to remodel the electrophysiological changes that occur during acute ischaemia by performing high-resolution optical mapping of transmembrane voltage. We also evaluated the capacity of UDCA to alter the expression and phosphorylation status of Cx43 using western blotting techniques. Finally, we used computational models of rat ventricular tissue to provide insights into the mechanisms of action of UDCA.

## Results

### Ischaemia–reperfusion induced ECG changes and arrhythmias

**Figure 1:**
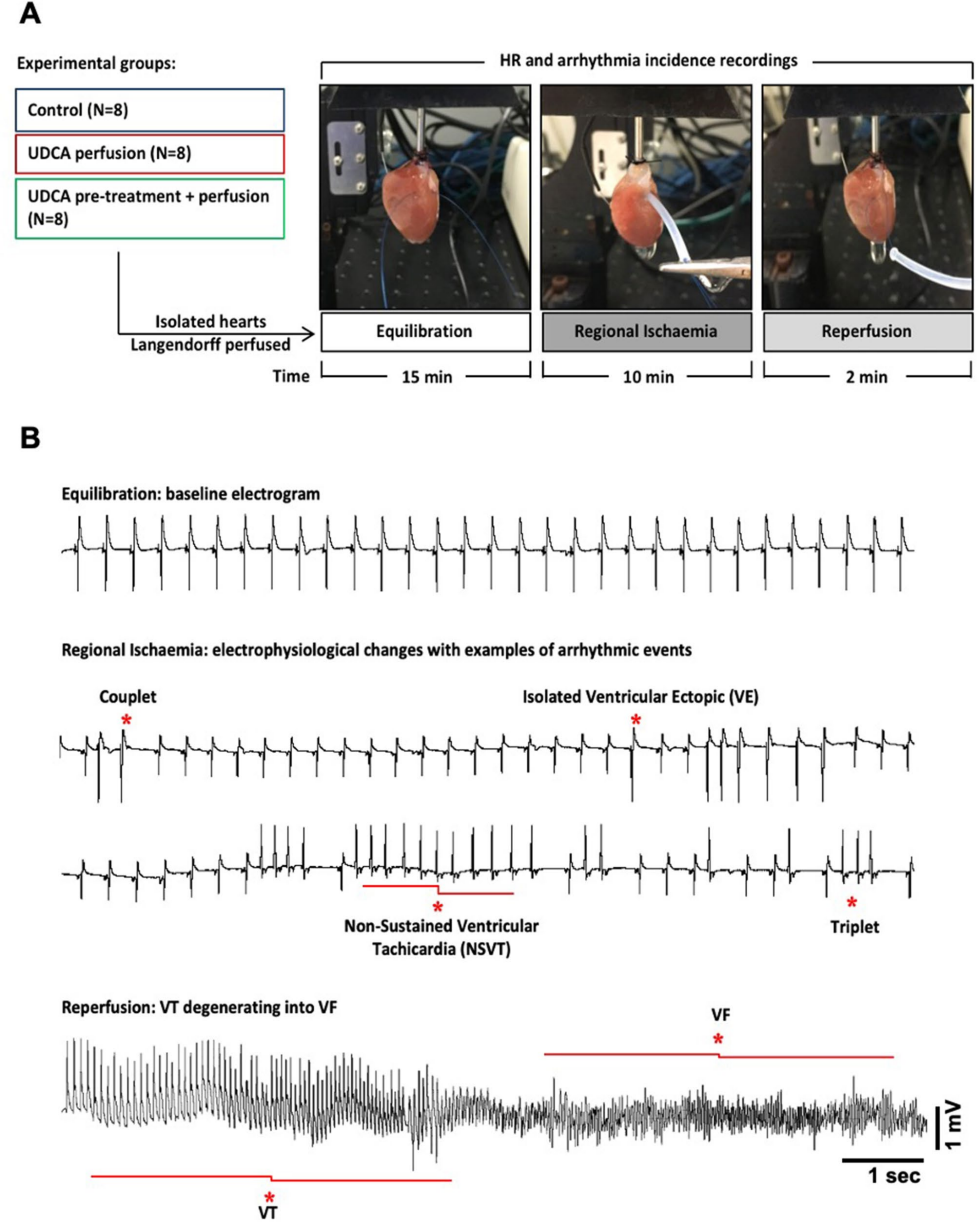
(**A**) Schematic experimental protocol of isolated rat hearts exposed to acute regional ischaemia for 10 min, followed by 2 min reperfusion. (**B**) Electrocardiogram recordings taken from the same heart showing marked electrophysiological changes and examples of ventricular arrhythmias occurred during the course of acute ischaemia in the form of isolated premature ventricular ectopic (VE), couplet, triplet and non-sustained ventricular tachycardia (NSVT), which result in ventricular tachycardia (VT), degenerating into ventricular fibrillation (VF) during reperfusion.

Successful induction of acute regional ischaemia was confirmed by changes in axis and morphology of unipolar electrograms (ECGs) recorded during the course of the experiments (representative ECGs in Fig. 1B), including ST-segment changes and T-wave abnormalities, accompanied by ventricular arrhythmic events such as isolated premature ventricular ectopic (VE) beat, couplet, triplet and non-sustained ventricular tachycardia (NSVT). After the ligature was released, ventricular tachycardia (VT) onset was observed within seconds of restoration of flow in some cases, generally degenerating into ventricular fibrillation (VF).

### Prolonged UDCA administration reduces ischaemia-induced arrhythmia incidence

**Figure 2:**
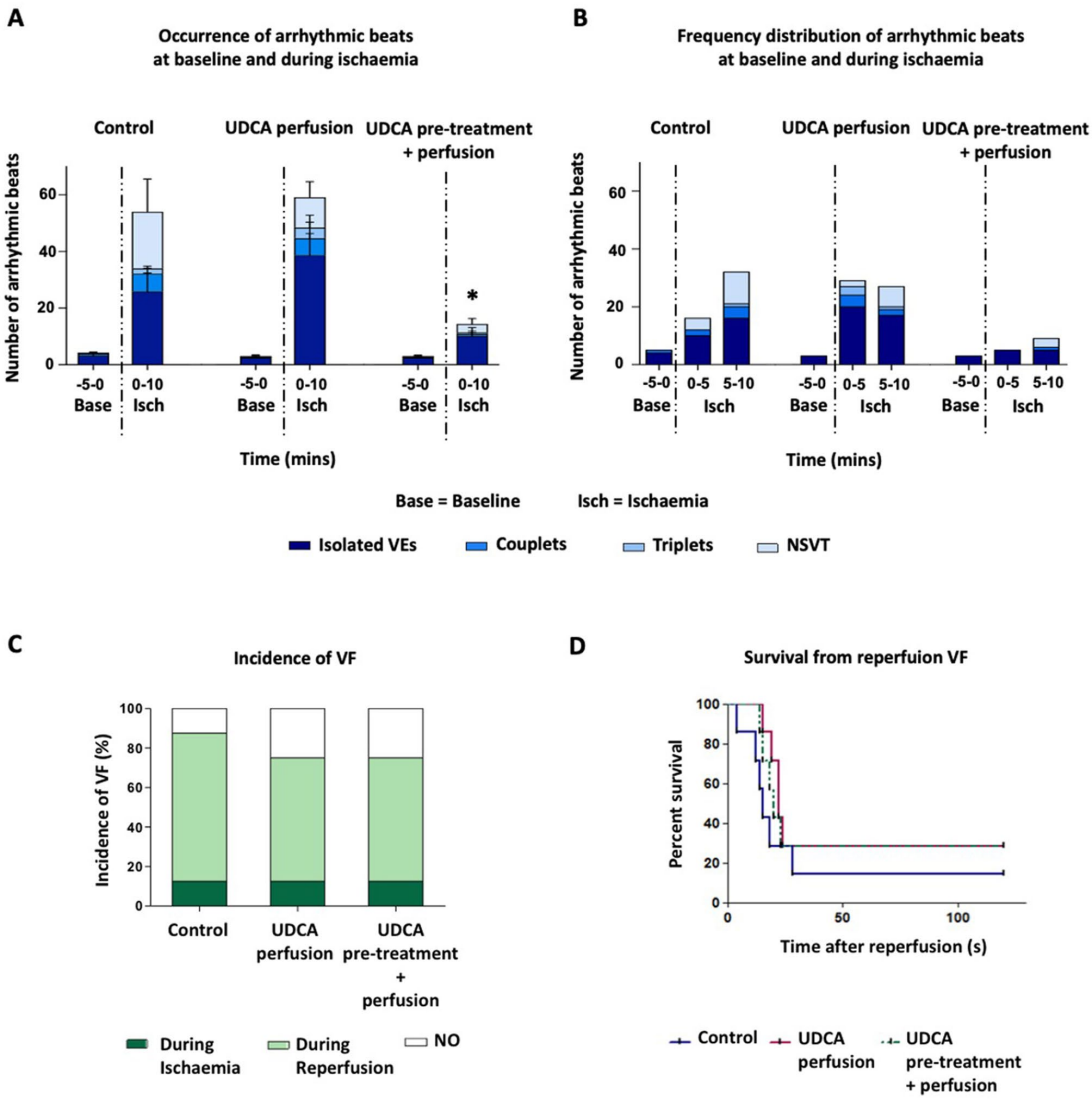
Summary of data on ischaemia–reperfusion studies. (**A**) Prolonged UDCA administration reduces ischaemia-induced arrhythmia incidence in the form of ventricular ectopic (VE) beats when the three experimental groups are compared during 10 min-induced acute myocardial ischaemia. (**B**) Distribution of the arrhythmic events at baseline and during ischaemia quantified in 5 min bins in the three experimental groups. (**C**, **D**) UDCA has no effect either on the incidence of VF during both ischaemia and reperfusion, or the onset time of the observed arrhythmias during reperfusion percent of survival when UDCA perfused (1 μM) and UDCA pre-treated and perfused (150 mg/kg/daily + 1 μM) groups are compared to control at different time points over the course of I–R studies. Data from control (n = 8), 1 μM UDCA (n = 8), and 150 mg/kg/daily + 1 μM UDCA (n = 8) hearts, *Isolated VEs significantly different from control (*p* = 0.036, by 1-way ANOVA (non parametric) test with post-hoc test).

No statistically significant difference was observed between control and acute UDCA perfused hearts (52 ± 16 and 58 ± 15, respectively) with respect to the total number of arrhythmic events occurring during 10 min-induced acute regional ischaemia (Fig. 2A). By contrast, there were significantly fewer (*p* = 0.028) ventricular arrhythmic beats between UDCA pre-treated (2 weeks) and perfused versus UDCA perfused hearts (10 ± 3 and 58 ± 15, respectively). This was driven by the significant decrease in VE activity occurring within the UDCA pre-treated and perfused hearts (10 ± 3), compared to the UDCA perfused hearts (39 ± 14) (*p* = 0.036). No temporal imbalance in their frequency distribution was observed between 0 to 5 and 5 to 10 min of ischaemia (Fig. 2B).

### UDCA has no effect on reperfusion-induced arrhythmia incidence

Figure 2C, D shows that, when reperfusion was induced for 2 min, all hearts exhibited increased vulnerability to arrhythmias in the form of VF when compared with ischaemia. The incidence of reperfusion VF was 75% in the control group with a mean onset time of 30 ± 15 s following reperfusion. UDCA treatment had no significant effect either on the incidence of VF during reperfusion, or the time of onset of reperfusion VF. Both UDCA perfused and UDCA pre-treated and perfused hearts had reperfusion VF incidences of 62.5% (5 of 8) and mean times to reperfusion VF of 49 ± 19 s and 47 ± 19 s for UDCA perfused and UDCA pre-treated and perfused hearts, respectively.

### Ischaemia-induced conduction velocity slowing is attenuated by prolonged UDCA administration, with no effect on action potential duration

**Figure 3:**
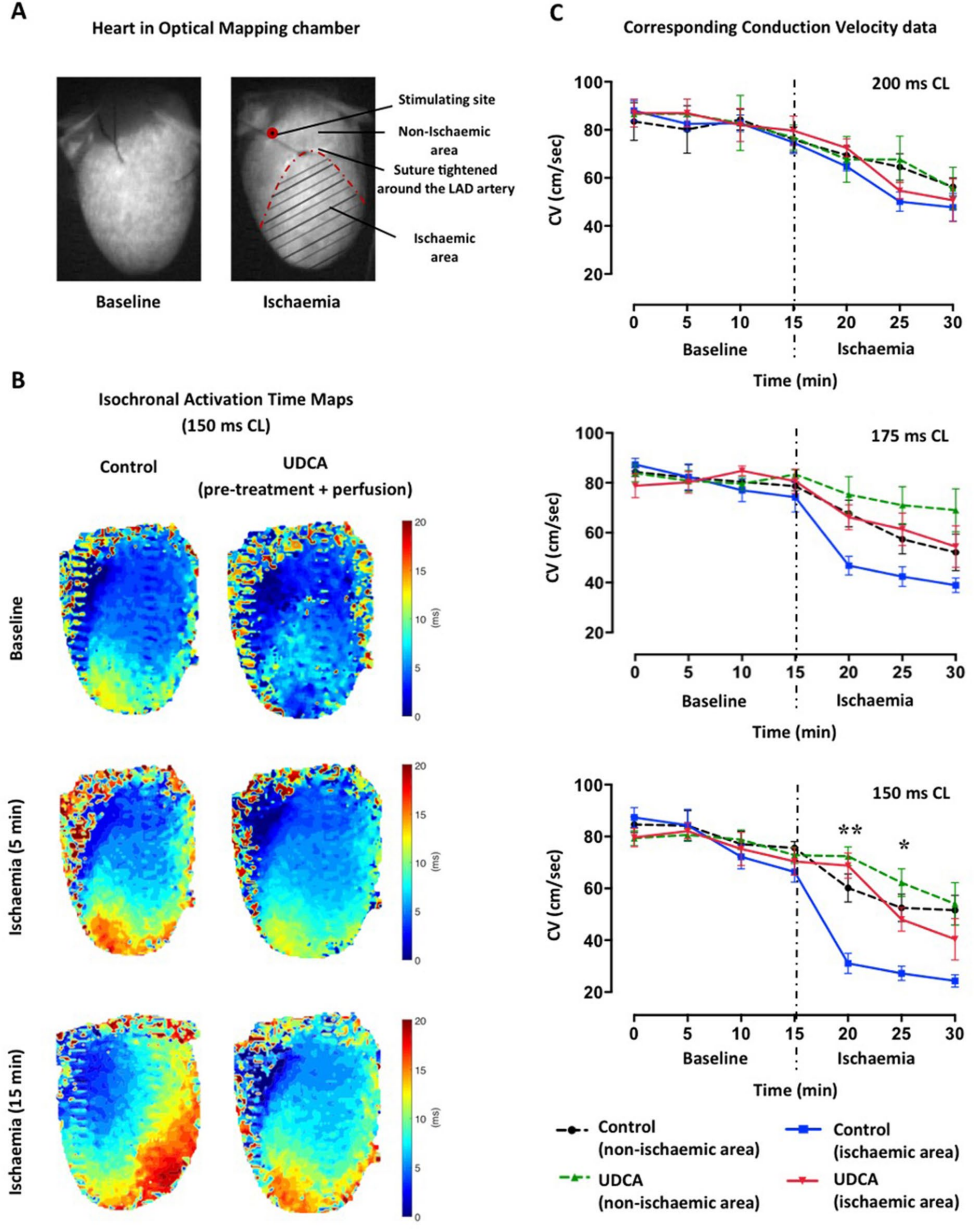
Summary of data on whole heart optical mapping. (**A**) Heart chamber set up at baseline and during myocardial ischaemia. (**B**) Representative isochronal activation time maps at baseline and during ischaemia of hearts pre-treated and perfused with UDCA, compared to control hearts when pacing at 150 ms cycle length (CL) (blue is early activation and red is late activation). (**C**) Corresponding conduction velocity (CV) data from non-ischaemic versus ischaemic areas, during sequential pacing at different CLs, showing that prolonged UDCA administration attenuates ischaemia-induced CV slowing in the early stage when pacing at 150 ms CL. Data from control (n = 6) and 150 mg/kg/daily + 1 μM UDCA (n = 5) hearts, ** , *CV from UDCA pre-treated and perfused hearts significantly different from control within ischaemic areas at 5 and 10 min ischaemia (**p = 0.0016, *p = 0.0255, by 2-way ANOVA (multiple comparisons) with post-hoc test).

**Figure 4:**
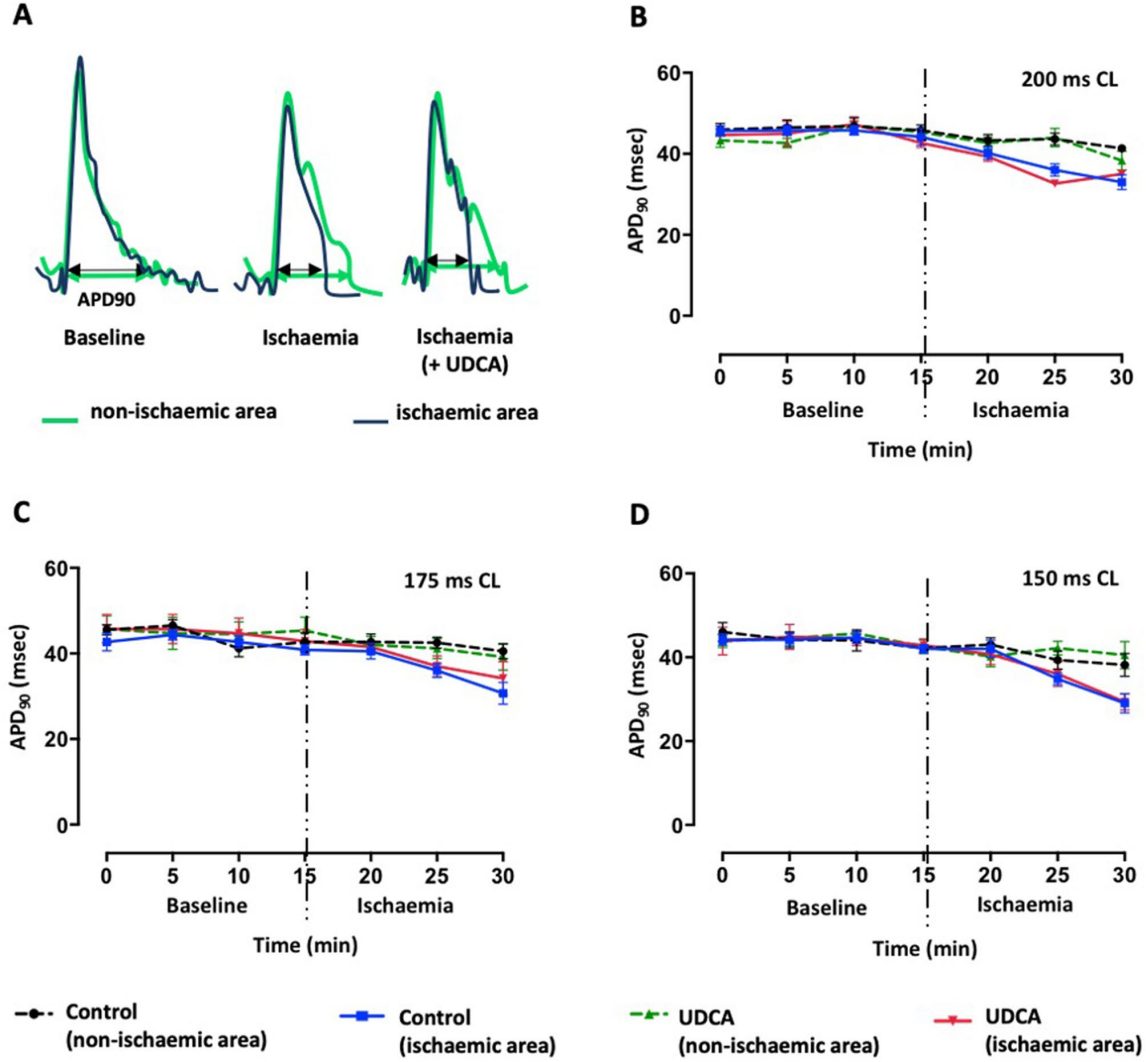
Summary of data on whole heart optical mapping. (**A**) Acute myocardial infarction causes action potential duration (APD)90 shortening. (**B**–**D**) Corresponding APD90 data from non-infarcted versus infarcted areas, at different pacing cycle lengths, showing no effect of prolonged UDCA administration against ischaemia-induced APD shortening. Data from control (n = 6) and 150 mg/kg/daily + 1 μM UDCA (n = 5) hearts.

UDCA pre-treated and perfused hearts were compared to control hearts with respect to CV from non-ischaemic versus ischaemic areas, at baseline and during ischaemia, when paced at the same cycle length (CL) (Fig. 3). UDCA attenuated ischaemia-induced CV slowing in the early stage (*p* = 0.0002), at shorter CL (pacing CL of 150 ms). In UDCA pre-treated and perfused hearts, CV was preserved within ischaemic areas (67 ± 8 cm/s, *p* = 0.0016; 50 ± 3 cm/s, *p* = 0.0255), compared to ischaemic areas in control hearts (33 ± 16 cm/s), for 5 and 10 min respectively, after ischaemia was induced. During the following 5 min period of ischaemia, CV progressively slowed within all groups. Acute ischaemia was also associated with progressive action potential duration 90 (APD_90_) shortening (Fig. 4), and UDCA treatment did not induce any significant changes in APD_90_ either at baseline or during ischaemia.

### Prolonged UDCA administration preserves Cx43 phosphorylation during acute ischaemia

**Figure 5:**
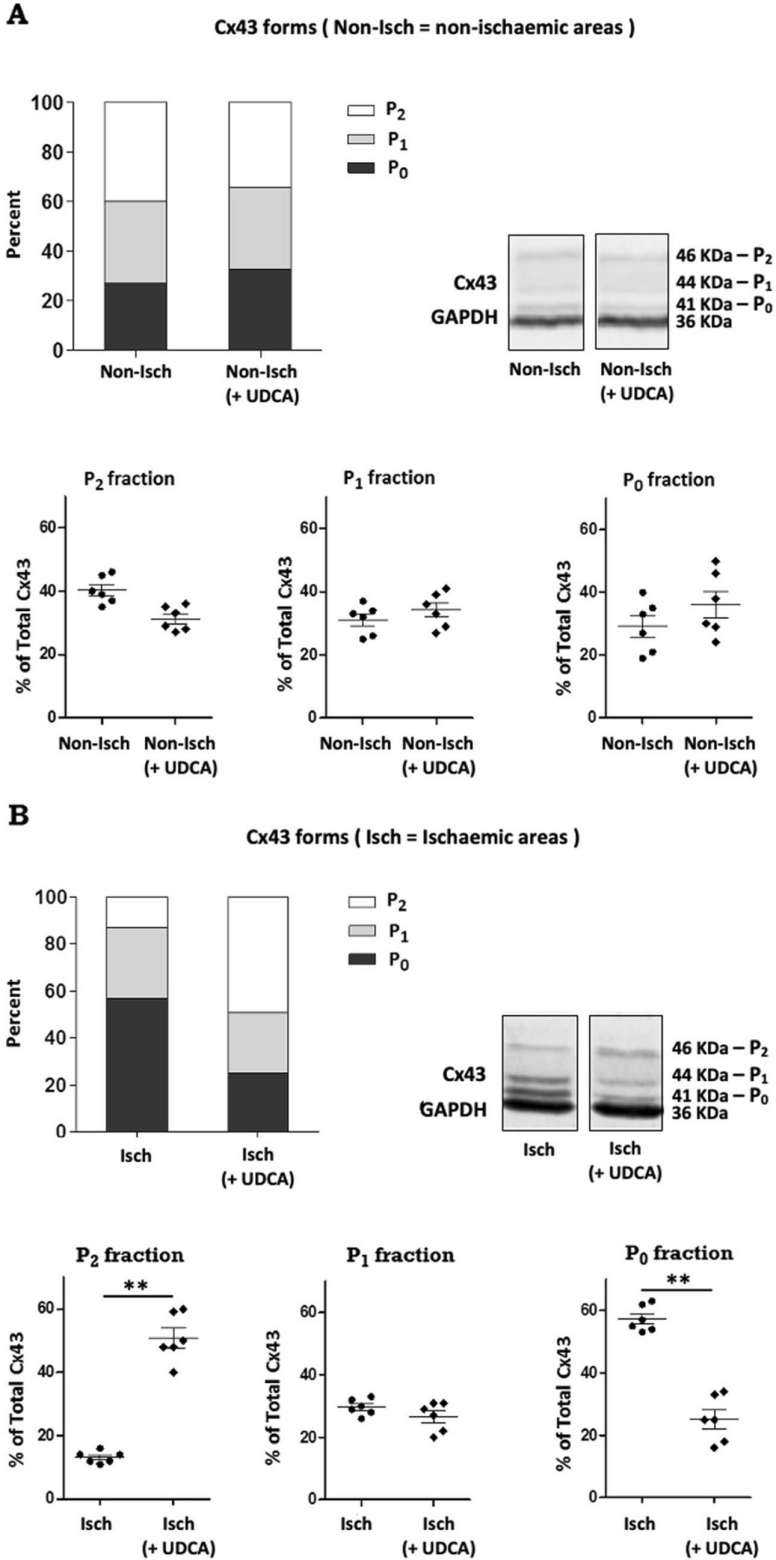
Western blot analysis of Connexin 43 expression from rat ventricle samples. Western blot detection and quantitative analysis of Connexin 43 (Cx43) from control and UDCA pre-treated and perfused hearts, comparing non-ischaemic (**A**) and ischaemic (**B**) areas. UDCA attenuated Cx43 dephosphorylation during 20 min ischaemia: increased P2 proportion and decreased P0 proportion for UDCA pre-treated and perfused hearts. The blots were cropped and full-length blots are available in Supplementary Fig. S1. Data from control (n = 6) and 150 mg/kg/daily + 1 μM UDCA (n = 6) hearts, **p = 0.0049, **p = 0.0022, by Student’s *t* test.

To investigate the effect of UDCA on Cx43 phosphorylation during acute ischaemia, we prepared immunoblots from control and UDCA treated hearts, comparing non-ischaemic and ischaemic regions (Fig. 5) (See Supplementary Fig. S1 for complete blots). Three different forms of Cx43 were detected when analyzed by sodium dodecyl sulfate polyacrylamide gel electrophoresis (SDS-PAGE), including a band at 41 kDa (corresponding to the fast migrating non-phosphorylated form and referred to as P0), and bands at 44 and 46 kDa (corresponding to slower migrating partially phosphorylated and phosphorylated forms commonly termed P1 and P2, respectively), consistent with previous reports showing that most of the Cx43 in the heart is phosphorylated. Acute ischaemia within the control experimental group was associated with an increase in the proportion of P_0_ non-phosphorylated Cx43 form (57 ± 1% compared with 27 ± 1% in the non-ischaemic tissue) and a corresponding loss of phosphorylated Cx43 (13 ± 3% compared with 40 ± 2% in the non-ischaemic tissue), which was prevented in UDCA treated hearts. There was greater phosphorylated Cx43 expression with UDCA treatment, compared to control (49 ± 2% and 13 ± 1% for UDCA treatment and control, respectively; *p* = 0.0049), and a corresponding decrease in P_0_ non-phosphorylated Cx43 (25 ± 1% and 57 ± 1% for UDCA treatment and control, respectively; *p* = 0.0022). No change in the total amount of Cx43 signal (phosphorylated plus non-phosphorylated forms) either under non-ischaemic or ischaemic conditions was observed.

### Mathematical modelling of effects of UDCA during ischaemia

**Figure 6:**
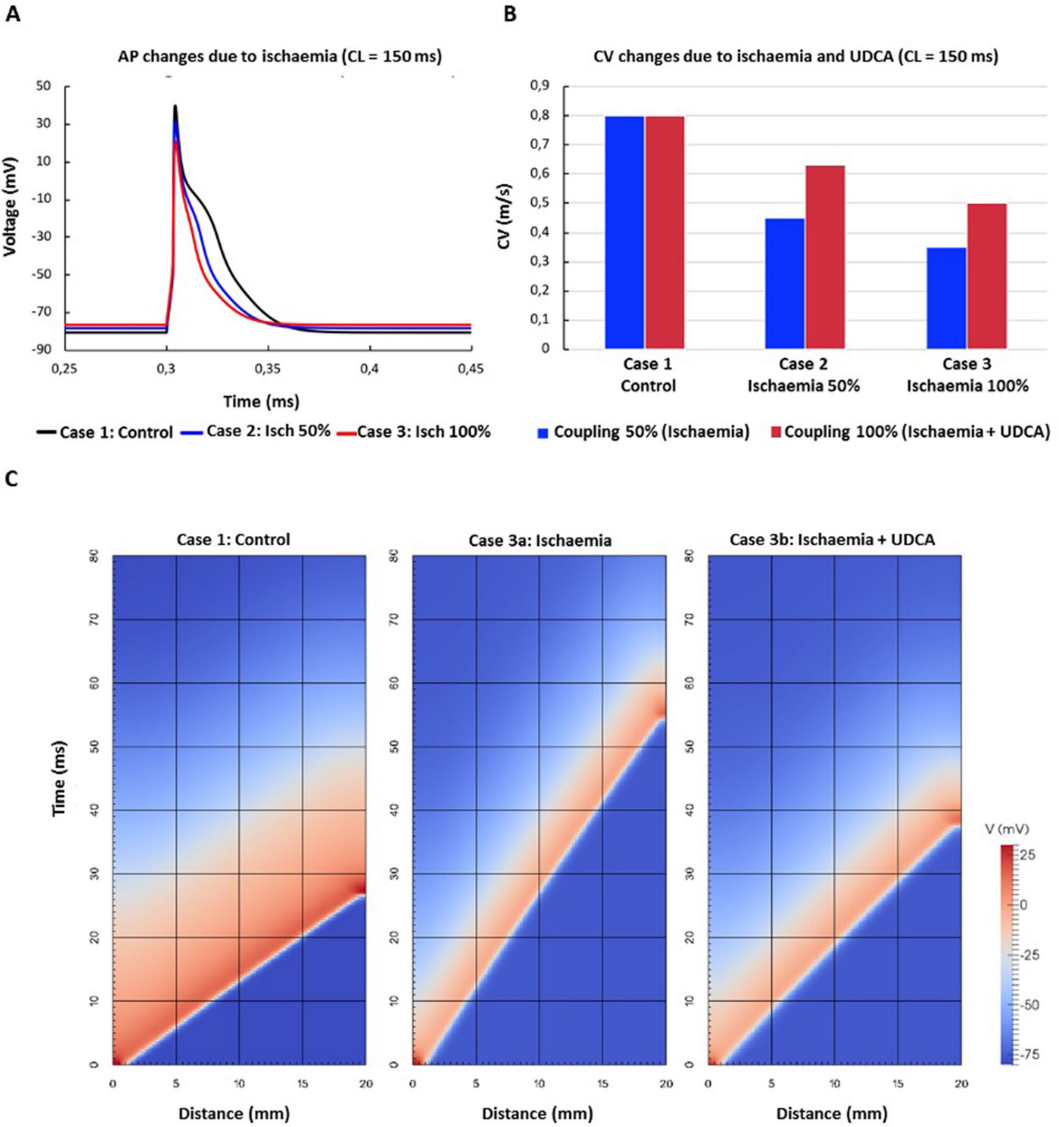
Effects of ischaemia and UDCA in mathematical models of rat ventricular cell and tissue. (**A**) Single cell action potential (AP) in the three cases considered (see “Results” section): control, 50% and 100% ischaemia. Ischaemia leads to progressive decreases of action potential duration (APD) and amplitude, and elevation of resting potential. (**B**) Conduction velocity (CV) in 2D rat ventricular tissue model in the same cases; in combination with ischaemia-induced reduction in cellular excitability, 50% reduction in the intercellular coupling (blue bars) leads to substantial decreases of CV; application of UCDA increases the coupling, and hence CV (red bars). (**C**) Space–time plots illustrating AP conduction along a scan line in 2D rat ventricular tissue model; 100% ischaemia (case 3a, see “Results” section) substantially slows down conduction, whereas an additional effect of UDCA (case 3b) partially restores the conduction, but not the cellular AP properties.

Figure 6A shows APs simulated with the rat ventricular cell model for three cases: (1) control, (2) 50% ischaemia, with *I*_Na_ reduced by 12.5%, *I*_CaL_ reduced by 25% and the resting potential depolarised by 1.9 mV, and (3) 100% ischaemia, with *I*_Na_ reduced by full 25%, *I*_CaL_ reduced by 50% and the resting potential depolarised by 3.8 mV. Ischaemia resulted in progressive decrease of APD_90_, in agreement with experiments (see Fig. 4).

In the respective tissue model, ischaemia cases (2) and (3) were simulated (a) with and (b) without an additional 50% reduction of the gap junctional coupling. Thus, cases (2a) and (3a) corresponded to ischaemia in the absence of UDCA, and cases (2b) and (3b) corresponded to ischaemia in the presence of UDCA that restores the phosphorylation of Cx43 (Fig. 5), and hence gap junctional coupling.

Figure 6B summaries the simulated values of CV for all cases considered. In the control case (1), CV has a value of 0.8 m/s that agrees with experimental data (Fig. 3). When ischaemia was introduced, CV sharply and progressively decreased to 0.45 m/s in case 2A and then 0.35 m/s in case 3A. This was due to the dual effect of 50% coupling reduction and decrease of the cellular excitability caused by the decreased inward currents and increased resting membrane potential. The additional UDCA effect—preservation of 100% coupling in ischaemia cases 2B and 3B—resulted in attenuation of ischaemia-induced CV slowing to 0.63 and 0.5 m/s, respectively, with a consequent increase in cardiac wavelength. These values were still lower than those in control, since the excitability remained partially suppressed by ischaemia.

## Discussion

In the present study, we explored the role of UDCA as an antiarrhythmic agent in the setting of acute regional I–R, supported by data from mathematical models of rat ventricular tissue. Our main findings were that, following prolonged administration, UDCA: (1) reduced ischaemia-induced arrhythmia incidence with no effect on reperfusion-induced arrhythmias, (2) attenuated ischaemia-induced CV slowing in the early stage with no effect on APD_90_ shortening, (3) maintained phosphorylation of Cx43 during acute ischaemia and (4) increased cardiac wavelength (WL) during acute ischaemia.

Acute myocardial ischaemia results in a spectrum of detrimental structural and electrophysiological effects, which in turn render the heart susceptible to potentially lethal ventricular arrhythmias. In an anaesthetised dog model described by Kaplinsky et al.^20^, ventricular arrhythmias occurred 2–10 min after coronary ligation, with a peak frequency at 5–6 min. In the present study, rat hearts subjected to acute regional ischaemia exhibited a high incidence in ventricular arrhythmias, mainly in the form of VEs, within the first 5 min of the onset of ischaemia, with a profile similar to that already reported in studies conducted by the Curtis laboratory in both conscious and anaesthetized rats as well as Langendorff-perfused rat hearts^21^. Prolonged UDCA treatment resulted in a significant reduction of VE beats, but did not reduce ischaemia-induced VT or VF, which both occurred very rarely in our model. The association between frequency of VEs and VT/VF is unclear, and may be related to the ventricular activation pattern rather than coupling interval^22^. Nonetheless, a reduction in the number of VEs with UDCA may reduce the overall probability of VT/VF being triggered by a critically-timed premature ectopic beat.

The observed antiarrhythmic activity of UDCA fits with a study conducted by Miragoli et al.^17^, showing the protective antiarrhythmic role of UDCA in an in vitro rat model of the cholestatic fetal heart, with our data suggesting that the effects of UDCA are not limited to fetal myocardium.

We also showed the importance of UDCA pre-administration with regards to its antiarrhythmic effects, not seen when acutely administered only. This could be attributed to the time needed to alter the gene expression profile to one that resists arrhythmogenesis. Bile acids are known to regulate the expression of genes involved in the synthesis and intestinal transport of bile acids such as 7α-hydroxylase, intestinal bile acid-binding protein and ileal sodium-dependent bile acid transporter. In the treatment of Intrahepatic Cholestasis of Pregnancy, a common liver disorder characterized by high circulating levels of bile acids in the maternal serum, the therapeutic effect of UDCA has been partially associated to the stimulation of hepatic canalicular transporter expression^23^. Pre-treatment with UDCA may exert its antiarrhythmic effects through as yet unknown effects on cardiac gene expression.

The high incidence in VF seen during reperfusion is in accordance with previous observations that ventricular arrhythmias can occur within seconds of restoration of blood flow to previously ischaemic myocardium^6,7^. Susceptibility to reperfusion arrhythmias is reported to be highly dependent on the duration of regional ischaemia prior to reperfusion, with a window of vulnerability which varies from species to species. For instance, for ex vivo experiments in rats, peak susceptibility occurs at 9–10 min, after which hearts fail to recover electrical activity during reperfusion and have presumably become irreversibly injured, which justifies the choice of a timescale of 10 min ischaemia for our I–R studies^24^. Our data are supported by existing studies in the literature, reporting that many drugs appear to have no effect against reperfusion-induced arrhythmias, despite being protective against ischaemia-induced arrhythmia, reflecting the different electrophysiological mechanisms underlying these two forms of arrhythmias^25^.

Increased diastolic Ca^2+^ overload and interstitial potassium, progressive intracellular acidosis and accumulation of amphipathic lipid metabolites during acute ischaemia promote pro-arrhythmic electrophysiological changes, including APD shortening and CV slowing^26–28^. Having established the antiarrhythmic benefits of prolonged administration of UDCA in a model of acute regional ischaemia, we performed optical mapping experiments to investigate the potential mechanisms through which UDCA protects against ischaemia-induced electrophysiological changes. We found that UDCA attenuates ischaemia-induced CV slowing during early ischaemia, although it has no effect on APD_90_ shortening. Similar results were presented in the study conducted by Miragoli et al*.*, where prolonged exposure to increasing concentrations of UDCA (from 0.1 to 100 μM) prevented arrhythmic events with the lowest concentration, which is close to the UDCA concentration used in the present study (1 μM), shown to maximally improve CV^17^. Further in agreement with our study, Adeyemi et al.^19^ also demonstrated that UDCA is able to prevent a significant reduction in CV slowing and arrhythmias in Langendorff-perfused neonatal hearts. This fetus-specific effect of UDCA was attributed to the capacity of UDCA to enhance calcium entry through *I*_Ca,T_, which is exclusively expressed in fetal hearts, and therefore not explored as potential UDCA target in the present study. Instead, given the well-established relation between ischaemia-induced CV slowing and Cx43 dephosphorylation, we focused mainly on Cx43 phosphorylation changes as the possible mechanism of underlying antiarrhythmic effects of UDCA.

Cx43 is the key protein making up the cardiac ventricular gap junctions of the heart, which are essential for fast and coordinated electrical impulse conduction. Cx43 is a phosphoprotein with clear evidence for phosphorylation at more than 12 serine (S) and tyrosine (Y) sites in the C-terminal tail of the protein via at least 6 kinases, and whose phosphorylated status is known to be dramatically modulated during ischaemia, most likely depending on the cellular ATP content^29,30^. Specifically, ischaemia results in a eightfold loss of pS365 and S325/328/330, which occurs rapidly (5 min), and a fivefold and 3.5-fold increase at S368 and S373, respectively^31–35^. Dephosphorylation of Cx43 results in uncoupling of gap junctions, therefore causing conduction slowing heterogeneities and facilitating arrhythmias^4,5^. Reduced Cx43 expression is reported to be directly linked to changes in CV and repolarization gradients^36^. In our studies, dephosphorylation of Cx43 under ischaemic conditions was prevented by prolonged administration of UDCA. This could be attributed to a modulatory effect on the cellular energetic competence. In support of our finding, there are a number of compounds whose antiarrhythmic effect has been associated with preservation of Cx43 in its phosphorylated status, and UDCA appears to be another such compound^37,38^. Most importantly, this finding provides a relevant mechanistic insight to our study. The effect of UDCA in maintaining Cx43 in a phosphorylated status during ischaemia explains, at least in part, the observed effect against ischaemia-induced CV slowing.

Cardiac WL is defined as the product of CV and APD and measures the distance traveled by an AP during the functional refractory period. Conditions that either shorten AP or slow conduction result in shorter WL, and thus facilitate the initiation and maintenance of reentrant arrhythmias^39^. The simulation results provide an explanation for the mechanism of UDCA action during ischaemia, which is via the restoration of intracellular coupling and CV, but not cellular excitability and APD. The UDCA-induced increase of CV (Fig. 6B) leads to proportional increase of WL (seen in Fig. 6C). From the simulations, WL can be estimated as 3.0 cm in the control case (1)—this is larger than the tissue size, making it more difficult for reentrant circuits to form, and reducing arrhythmogenicity. In ischaemic cases (2a) and (3a), WL is reduced to 1.2–1.8 cm, which is smaller than the tissue size and allows for reentrant circuits to form. In cases (2b) and (3b), additional UCDA effects increase WL to 2.0–2.5 cm, which can explain the reduction in arrhythmogenicity. Ischaemia-induced VEs have been shown to be due in part to reentrant mechanisms involving intramural reentry^40^. Pogwidz et al. assessed the mechanisms responsible for the initiation and maintenance of premature ventricular complexes (PVCs) and VT during early ischaemia, and showed that in 76% of cases, initiation of single PVCs and the first beat of VT occurred through intramural reentry. The increase in wavelength with UDCA would be likely to contribute to the reduction in VEs during ischaemia, by reducing reentry. It is also possible that UDCA may reduce ischaemia-induced ectopy via effects on non-reentrant mechanisms, such as via the suppression of triggered activity via affecting Na and Ca or HCN currents, though this was outside the scope of our study.

The isolated perfused heart preparation is a well-established method in cardiovascular research to study I-R injury and the effect of drugs on arrhythmia incidence. However, the ex vivo model has limitations and does not recapitulate all of the features of acute ischaemia in vivo, including the lack of neurohormonal input, and our results need to be interpreted within this context.

In conclusion, our study shows that prolonged UDCA administration reduces the incidence of arrhythmias during acute ischaemia. Its antiarrhythmic effects may be mediated in part by maintaining Cx43 in a phosphorylated status, thereby preserving ventricular electrical uncoupling and CV slowing, which in turn result in increased WL. The potential antiarrhythmic effects of prolonged UDCA administration merit further investigation. The natural progression and future direction of this work would indeed be to determine the relevance of our findings in well designed in vivo experiments. This is an important step towards the validation of prolonged administration of UDCA as novel therapeutic strategy to reduce ventricular arrhythmias due to ischaemic insults in patients with a history of coronary artery disease.

## Methods

### Animals

Twenty-four male Sprague–Dawley rats (250–300 g) were used to perform ex vivo I-R studies, and randomly assigned to the following three groups: (1) untreated control [rats fed on a normal diet]; (2) acute UDCA treatment [untreated rats whose heart was excised and perfused ex vivo with UDCA (1 µM)]; (3) prolonged UDCA treatment [rats receiving UDCA (150 mg/kg/daily) suspended in 1% ethanol in the drinking water, administered for 2 weeks before the heart was excised, and perfused ex vivo with UDCA (1 µM)]. The drug concentrations chosen have been reported to have cardiac effects in rats^17,41^. A further twenty-three animals were assigned to group 1 and 3 only and used for optical mapping and western blotting studies.

### Ex vivo ischaemia–reperfusion

Isolated hearts were subjected to acute ischaemia according to the protocol illustrated in Fig. 1A, as previously described^24^. Briefly, animals were placed in an induction chamber and anaesthetised by inhalation of isoflurane (Abbott, USA) (5% in oxygen). When a sufficient depth of anaesthetisation was achieved (confirmed by loss of righting ability and pedal pinch reflex), rats were sacrificed by cervical dislocation, the hearts excised and rapidly perfused ex vivo on a Langendorff apparatus at a fixed coronary flow rate (CFR) of 15 ml/min with Krebs–Henseleit buffer solution (in mmol/L: NaCl 118.5, CaCl_2_ 1.85, KCl 4.5, D-Glucose 11.1, NaHCO_3_ 25.0, MgSO_4_ 2.5, NaH_2_PO_4_ 1.4) at 37 °C and equilibrated with a gas mixture of 95% O_2_ and 5% CO_2_ (pH 7.4). Hearts were perfused either with control perfusate or 1 µM UDCA, as above. A 15 min stabilisation period was allowed before acute regional ischaemia was induced by left anterior descending (LAD) artery ligation. 10 min after LAD ligation, the ligature was released, and the myocardium reperfused for the following 2 min. For optical mapping studies, hearts were only subjected to acute regional ischaemia with no reperfusion, based on the results obtained from I-R studies. A longer duration of ischaemia (20 min) was selected for the optical mapping experiments because of the slower development of ischaemia-induced electrophysiological changes due to reduced energy consumption, with the use of the excitation–contraction uncoupler blebbistatin.

Unipolar ECGs were continuously recorded by placing a silver unipolar electrode at the right ventricle, while the reference electrode was attached to the aortic cannula. Both electrodes were connected to a Bioamplifier and PowerLab data acquisition system (AD Instruments, Sydney, Australia), and the signals recorded at a sampling frequency of 1 kHz and displayed using LabChart 8 software (AD instrument). Heart rate (HR) and arrhythmia incidence during both ischaemia and reperfusion periods were analysed from the ECG recordings. Hearts were excluded from the study if demonstrating any contractile dysfunction or other evidence of instability (e.g. HR lower than 200 bpm, ventricular arrhythmias) at baseline. Arrhythmias were classified according to the Lambeth Conventions Guidelines^42^.

### Whole heart optical mapping of transmembrane voltage

Optical mapping of transmembrane voltage was performed as previously discussed^24,43,44^. Hearts were firstly perfused and stabilized on a Langendorff apparatus as described above. Following the initial 15 min of stabilization, hearts were stained with the voltage-sensitive fluorescent dye (N-(4-Sulfobutyl)-4-(6-(4(Dibutylamino)phenyl)hexatrienyl)Pyridinium [RH237; (1 mg/ml) in dimethyl sulfoxide (DMSO); Thermo-Fisher; Massachusetts, USA], and perfused with the excitation–contraction uncoupler blebbistatin (10 μM; Tocris Bio-Sciences; Cambridge, UK) to eliminate motion artefacts during the experiment. Four 530 LEDs were used to excite the epicardial surface of the heart exciting RH237, of which the emitted light was collected and passed through a 620 LP filter. Signals were recorded during pacing from the base of the left ventricle (pacing CL of 200–175–150 ms) using a MicroPace stimulator (MicroPace, Auckland, New Zealand), for 2 s at a time at regular intervals, before and after ischaemia was induced as above for 20 min.

Optical mapping data was acquired and displayed using the CardioPlex software (RedShirt Imaging), and activation patterns were reconstructed by using custom MATLAB code (MathWorks, Cambridge, UK). Data analysis includes recordings from the first 15 min only as the data were not analyzable at 20 min ischaemia time point. Isochronal maps and CV were calculated as previously described^45^. APD_90_ was calculated as the duration between the activation time and time of 90% repolarization.

### Protein extraction and western blotting

Hearts subjected to a 20 min period of coronary artery occlusion with no reperfusion were removed from the perfusion apparatus, samples snap-frozen in liquid nitrogen within a few minutes of collection and stored at − 80 °C for analysis. From each heart, two segments were frozen for Cx43 immunoblotting: ischaemic anterior left ventricle (ALV) and non-ischaemic ALV. Pulverised frozen samples (20 mg) were suspended in 0.1 mg/μl SB_20_ solution and sonicated (1 min approximately). After addition of 2-mercaptoethanol (2.5% final concentration), proteins (50 μg) fractionated on 8% SDS-PAGE were electrophoretically transferred to 0.2 μM polyvinylidene difluoride (PVDF) immunoblot membranes (BIO-RAD). After 1 h of incubation in TBS-Tween blocking solution (in mM: 100 Tris-HCl, 150 NaCl and 0.1% tween-20) containing 5% skimmed milk, membranes were probed with Anti-Cx43 (diluted 1:500, AB1727, Merck Millipore, UK) overnight at 4 °C. Blots were then incubated for 1 h with the appropriate goat anti-mouse alkaline phosphatase-conjugated secondary antibody (diluted 1:2,500, ThermoFisher), and developed with a 5-bromo-4-chloro-3-indolyl phosphate (BCIP)/nitro blue tetrazolium (NBT) substrate solution for 10–30 min until color development. The band intensity was assessed using ImageJ software and referenced to GAPDH (diluted 1:1,000, Invitrogen).

### Mathematical modelling

The dynamics of cardiac AP can be described by a Hodgkin-Huxley type equation: ${C}_{\mathrm{m}}dV/dt=-{I}_{\mathrm{ion}}$, where *V* (mV) is the membrane potential, *t* (s) is time, *C*_m_ (pF) is the cell membrane capacitance, and *I*_ion_ (pA) is the total membrane ionic current. A detailed cell electrophysiology model has been developed to describe this current (and hence, cell-specific AP properties), in healthy rat ventricular myocytes^46^. In ischaemic myocytes, the conductances of several ionic currents in this model were modified to account for the known cellular effects of ischaemia (such as acidosis), as described previously^47,48^. Specifically, the fast sodium current, *I*_Na_, was reduced by 25%, the L-type calcium current, *I*_CaL_, was reduced by 50%, and the resting potential was depolarised by 3.8 mV. These changes resulted in about 20% decrease of APD and 10% increase of AP amplitude, in agreement with our experimental recordings (see “Results” below).

Ventricular tissue composed of electrically coupled myocytes was modelled using the standard monodomain equation: $dV/dt=D\Delta V-{ I}_{\mathrm{ion}}/{C}_{\mathrm{m}}$, where $\Delta$ is the Laplacian operator and *D* (mm^2^/ms) is the diffusion coefficient that characterizes intercellular coupling via gap junctions^47,48^. In case of ischaemic tissue, the standard value of *D* was reduced by 50% to account for the known ischaemia-induced decrease of gap junctional coupling and CV. Note that both cellular (acidosis) and tissue (coupling reduction) effects of ischaemia develop within 10 min of its onset^5,49^, which is similar to the timescale considered in our experiments. Other effects of ischaemia (such as hyperkalaemia) were not measured in the experiments, and hence were not included into the model.

### Statistics

Statistical analysis was conducted using GraphPad Prism 8 (GraphPad Software Inc.), and results are presented as mean values ± SEM. A one/two-way analysis of variance (ANOVA) was first carried out to test for any differences between multiple groups, and appropriate post-hoc tests performed if ANOVA was significant. Multiple comparisons between groups were made with Tukey tests. Comparisons between two groups were performed using unpaired Student’s *t* test. For all tests, *p* < 0.05 was considered significant.

### Ethical approval

The investigation was performed in accordance with standards set out in the United Kingdom Animals (Scientific Procedures) Act 1986, approved by Imperial College London Ethical Review Board and carried out under Project License PPL PEE7C76CD.

## Supplementary information

Supplementary Information.
