# Supplementary material for: Prolonged ursodeoxycholic acid administration reduces acute ischaemia-induced arrhythmias in adult rat hearts
Source: Sci Rep. 2020 Sep 17;10:15284. doi: 10.1038/s41598-020-72016-4 (PMC7499428; doi:10.1038/s41598-020-72016-4)
Supplement: Supplementary file 1 — Supplementary Information. [file 41598_2020_72016_MOESM1_ESM.docx]

**Prolonged ursodeoxycholic acid administration reduces acute ischaemia-induced arrhythmias in adult rat hearts**

Elisa Ferraro, MRes^1^, Lidia Pozhidaeva, PgD^2^, David S. Pitcher, PhD^1^, Catherine Mansfield, PhD^1^, Jia Han Benjamin Koh, BEng^2^, Catherine Williamson, PhD^3^, Oleg Aslanidi, PhD^2^, Julia Gorelik, PhD^1*^ and Fu Siong Ng, MRCP, PhD^1*^

^1^National Heart and Lung Institute, Imperial College London, London, United Kingdom

^2^School of Biomedical Engineering and Imaging Science, King’s College London, London, United Kingdom

^3^Department of Women & Children’s Health, King’s College London, London, United Kingdom

*Joint senior/corresponding authors

**Supplementary information figure legends**

**Supplementary information, Fig. S1** Western blot analysis of connexin 43 expression from rat ventricle samples. Uncropped western blots of Connexin 43 (Cx43) from untreated and UDCA pre-treated and perfused hearts, comparing non-ischaemic **(A)** and ischaemic **(B)** areas. UDCA attenuated Cx43 dephosphorylation during 20 minutes ischaemia: increased P_2_ proportion and decreased P_0_ proportion for UDCA pre-treated and perfused hearts. Red boxes denote the regions of the blots used in Figure 5.

**Supplementary information, Fig. S2** Heart rates from ischaemia-reperfusion studies. Graph showing the sequential change of heart rate (HR) between control, UDCA perfused (1 μM) and UDCA pre-treated + perfused (150 mg/kg/daily + 1 μM) groups (n=8 for each group) at different time points over the course of ischaemia-reperfusion studies. 0-15 mins refers the points of time as minutes before ischaemia was induced for a period of 10 minutes, followed by reperfusion. HR is expressed as beats per minute (bpm).

**Supplementary information figures**

**
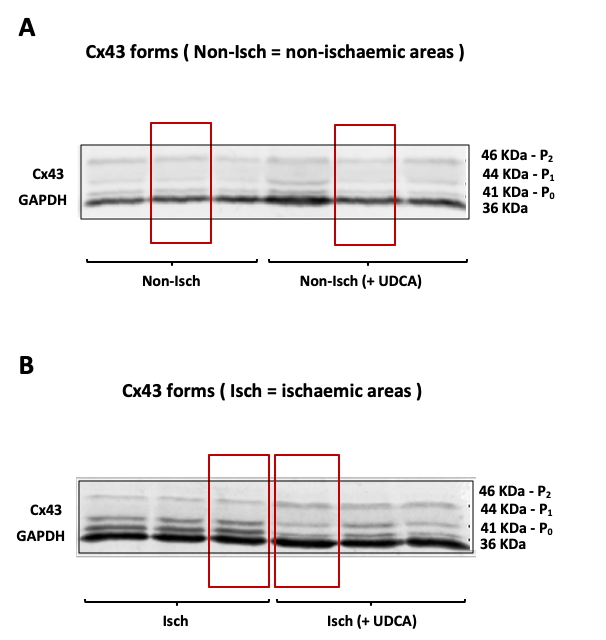
Supplementary information, Fig. S1**

**Supplementary information Fig. S2**

**
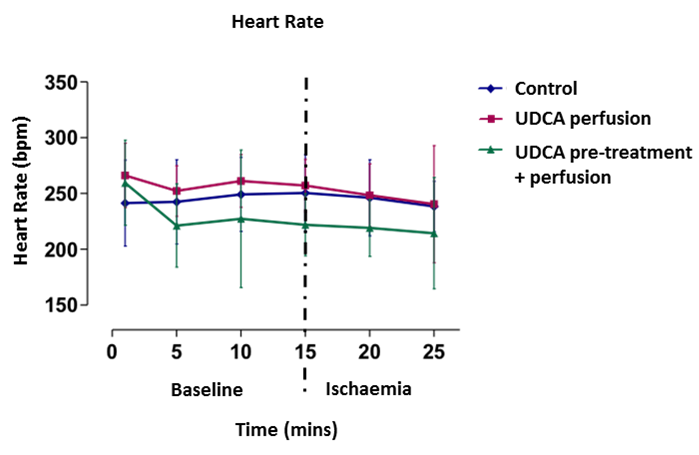
**

**Supplementary information results**

**Ischaemia-reperfusion hemodynamic-induced changes**

**Supplementary Fig. S2** illustrates HR values from the three groups analyzed. Within the control group, HR remained constant over the course of the experiments: at baseline, immediately at the beginning and at the end of the 10-minute period of coronary artery occlusion, HR values were 241±14; 250±12 and 238±9 bpm respectively. UDCA treatment did not affect HR over the course of the experiments. Similar to control hearts, at the same time points, HR values for UDCA perfused hearts were 266±10; 257±8 and 240±20 bpm respectively, while for UDCA pre-treated and perfused hearts HR was 260±13; 222±10 and 214±19 bpm respectively. No statistically significant difference was observed when the three groups were compared, at different time points over the course of the I-R studies.

**Supplementary information discussion**

**UDCA does not affect HR in isolated-perfused rat hearts undergoing acute I-R**

HR is a well-established determinant of arrhythmias susceptibility, with higher HR predisposing to arrhythmias.^1^ In the present study, no statistically significance difference was observed between the groups (**Supplementary Fig. S2**), and the overall HR stability suggested that all hearts were in good health. Therefore, it is unlikely that HR would have a role in arrhythmias susceptibility. The low HR values over the course of the stabilization period preceding ischaemia were of concern as Sprague-Dawley rats HR is known to range between 350 and 450. One possible explanation is the well documented high sensitivity of HR to temperature. Accordingly, when whole heart optical mapping was performed, it was noticed that HR tent to increase, probably due to the use of the perspex optical mapping chamber, which preserved the heart from temperature fluctuations. Despite lower than usual, HR values were still in accordance with the exclusion criteria applied to Langendorff perfused hearts with regard to rat HR.^2^

**Supplementary information references**

1. Bernier, M., Curtis, M.J. & Hearse, D.J. Ischemia-induced and reperfusion-induced arrhythmias: importance of heart rate. *Am J Physiol* **256**(1), H21-31 (1989).
2. Bell, R.M., Mocanu, M.M. & Yellon, D.M. Retrograde heart perfusion: the Langendorff technique of isolated heart perfusion. J M Cell Cardiol **50**(6), 940-50 (2011).
